# Construction and efficacy evaluation of a model for early diagnosis of pediatric sepsis based on LASSO-logistic regression

**DOI:** 10.3389/fped.2025.1624278

**Published:** 2025-08-26

**Authors:** Yan Jiang, Weikai Wang, Ruifeng Xu, Chen Wang, Zhongtao Wang, Xin Wang, Jingguo Zhang, Yanxia Wang

**Affiliations:** ^1^ Pediatric Emergency Center, Gansu Provincial Maternity and Child-Care Hospital (Gansu Provincial Central Hospital), Lanzhou, China; ^2^ Clinical Research Center, Gansu Provincial Maternity and Child-Care Hospital (Gansu Provincial Central Hospital), Lanzhou, China; ^3^ Department of Pediatrics, Gansu Provincial Maternity and Child-Care Hospital (Gansu Provincial Central Hospital), Lanzhou, China; ^4^ The First Clinical Medical College, Gansu University of Chinese Medicine, Lanzhou, China

**Keywords:** sepsis, pediatric, diagnosis, models, regression analysis

## Abstract

**Objective:**

The aim of this study was to analyse the clinical characteristics and related risk factors of Pediatric Sepsis, construct a column-line diagram model to predict the likelihood of Pediatric Sepsis, and validate the model to facilitate primary care paediatricians to quickly and quantitatively assess the risk of Pediatric Sepsis.

**Methods:**

This single-center retrospective study included children hospitalized for infections at Gansu Provincial Maternity and Child-Care Hospital from January 2018 to June 2024. Data on 39 variables covering baseline characteristics, vital signs, and laboratory indicators were collected. The samples were randomized into training and validation groups in a 7:3 ratio. Least Absolute Shrinkage and Selection Operator (LASSO) regression was used for initial data screening and dimensionality reduction, followed by Logistic regression to identify independent risk factors for sepsis. Predictive modeling was then performed. The performance of the column-line plots was internally validated using ROC curves, calibration curves, and decision curve analysis (DCA).

**Results:**

The development dataset included 834 patients with severe infections, of whom 212 (25.4%) developed sepsis. Seven predictors were identified as independent risk factors: respiratory rate, temperature, immature granulocyte percentage, platelets, procalcitonin, fibrinogen, and lactic acid. A predictive column-line diagram was created using these predictors. Internal validation showed that the column-line diagrams had good discriminatory ability, calibration, and clinical applicability.

**Conclusion:**

A column-line diagram was successfully developed to predict the incidence of sepsis in children using seven commonly used clinical and laboratory indicators. The model demonstrated good performance and clinical validity through internal validation.

## 1 Introduction

Children possess immature immune systems that render them vulnerable to infectious illnesses (1, 2). In low- and middle-income nations, the mortality rate associated with Pediatric Sepsis can reach 25%, posing a significant hazard to children's well-being and survival (3).

Sepsis is characterized as a life-threatening dysfunction of organs resulting from an imbalanced host response triggered by an infection (4). The pathogenesis of sepsis is complex, and until the year 2020, clear diagnostic criteria for early pediatric sepsis were lacking. The publication of the International Guidelines of the Campaign to Save Sepsis in 2020 provided a framework for diagnosing sepsis in children (5), stipulating that a Pediatric Sequential Organ Failure Assessment (PSOFA) score of ≥2 in patients with infections or suspected infections indicates sepsis. However, this guideline is subject to limitations, as the PSOFA score is derived from the Sequential Organ Failure Assessment (SOFA) score utilized in adults. Children cannot be considered as miniature versions of adults, hence a straightforward application of adult criteria to assess organ function in children may not be precise (2). The onset and progression of sepsis entail specific temporal dynamics and alterations. Children with sepsis may not exhibit typical septic presentations initially (6), thereby complicating early diagnosis.

Inadequate understanding of sepsis among some pediatric practitioners and the prevalent issue of delayed diagnoses underscore the ongoing challenge of enhancing early sepsis detection through routine laboratory assessments. There is a pressing need for a predictive model capable of accurately discerning individuals at risk of sepsis progression, enabling the prompt administration of standardized treatments to mitigate mortality rates. In this study, we conducted a retrospective analysis of laboratory findings from children with and without sepsis, and developed and validated a composite model utilizing Least Absolute Shrinkage and Selection Operator (LASSO) regression and multifactorial Logistic regression, which achieved favorable results.

## 2 Materials and methods

### 2.1 Study population and design

**Figure 1:**
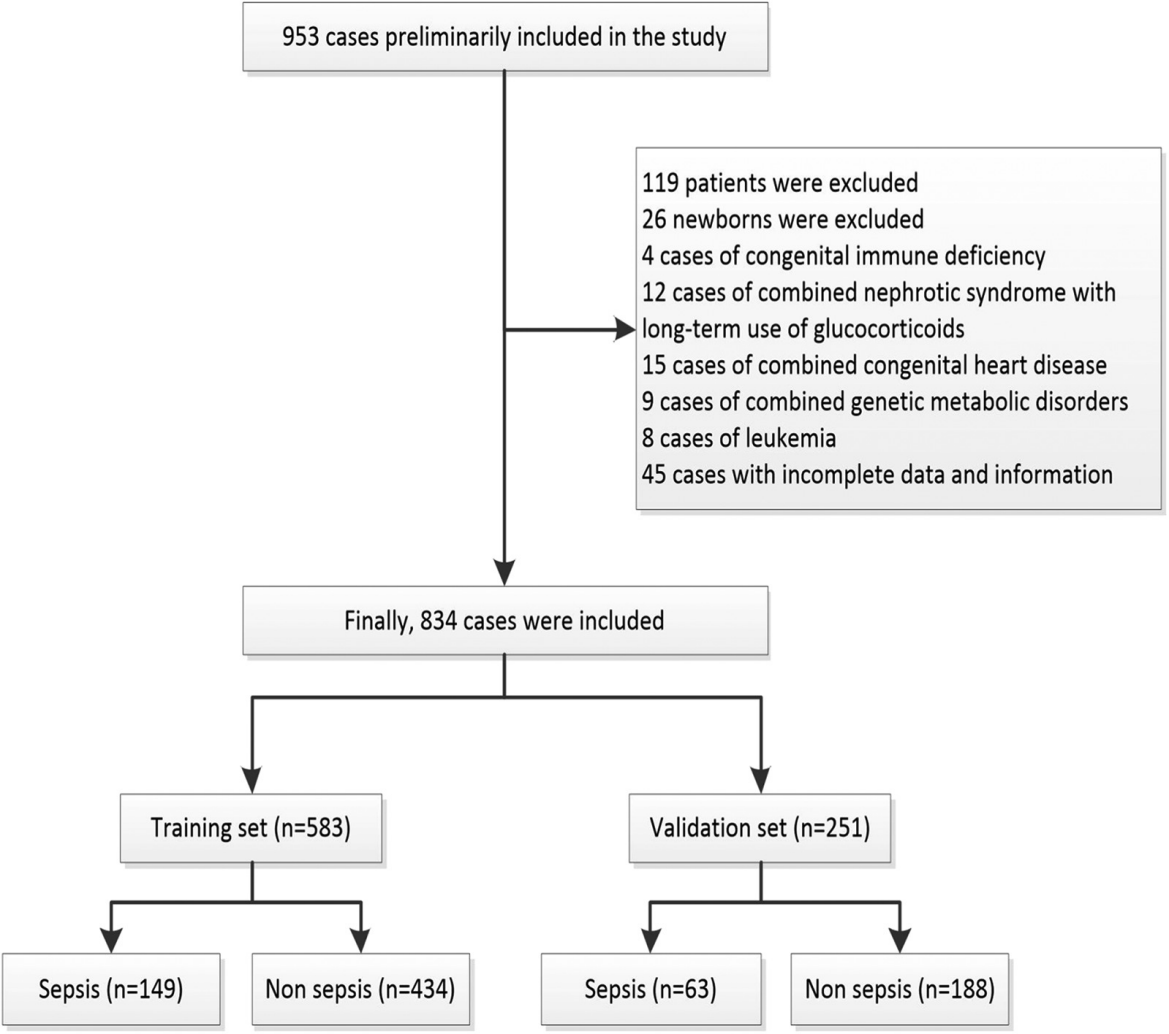
Enrolment and allocation of study participants. Flowchart depicting the enrolment of 953 patients, exclusion, and eventual inclusion of 834 patients in the training set (*n* = 583) and validation set (*n* = 251) of the predictive model development.

This study was conducted at a single tertiary hospital in Gansu, China, focusing on children hospitalized for infections in 13 different subspecialty pediatric departments of the Gansu Provincial Maternity and Child-Care Hospital (Gansu Provincial Central Hospital) between January 2018 and June 2024. The inclusion criteria comprised children aged 1 month to 14 years with complete case data. Exclusion criteria included children with advanced malignant tumors, congenital immunodeficiency, recent use of immunosuppressive drugs, severe combined congenital diseases, and those with missing clinical data. All enrolled patients were divided into sepsis group and non-sepsis group based on the presence or absence of sepsis. The diagnostic criteria for the sepsis group were defined as infection or suspected infection + PSOFA score ≥2. Of the initial 953 cases, 119 were subsequently excluded, resulting in a final cohort of 834 cases. These 834 infected patients were randomly divided into training and validation sets, with the training set comprising 583 cases (149 septic and 434 non-septic children) and the validation set consisting of 251 cases (63 septic and 188 non-septic children). Details of the data screening process are depicted in Figure 1.

This retrospective study solely entails the collection and analysis of existing data without influencing the treatment of any patients. The program has been reviewed and approved by the Ethics Committee of Gansu Provincial Maternity and Child-Care Hospital (Gansu Provincial Central Hospital) with the ethical filing number: (2023) GSFY Lun Audit (11).

### 2.2 Selected predictor variables

The researchers utilized a scientific research platform to gather pertinent clinical data of patients. All vital signs and laboratory tests were obtained within 24 h of hospital admission. encompassing: (1). fundamental information: age, gender, weight, ethnicity, nutritional status, and site of primary infection, etc; (2). vital signs: temperature (T), heart rate (HR), respiratory rate (RR), systolic blood pressure (SBP), diastolic blood pressure (DBP), differential pulse pressure (PP), and mean arterial pressure (MAP); Because the fluctuation range of respiratory rate and heart rate varies in children of different ages, respiration and heart rate were grouped according to the normal fluctuation range of different age groups, including below normal, normal, and above normal levels. The specific criteria for such divisions were derived from Chinese and American pediatric basic critical care support training materials (7) (Table 1). 3. Laboratory indices: white blood cells (WBC), haemoglobin (Hb), platelets (PLT), percentage of neutrophils (*N*%), percentage of lymphocytes (L%), immature granulocyte percentage (IG%), C-reactive protein (CRP), procalcitonin (PCT), albumin (ALB), alanine aminotransferase (ALT), aspartate aminotransferase (AST), Total bilirubin (TBIL), creatinine (Cr), urea nitrogen (BUN), cystatin C (CYSC), creatine kinase (CK), potassium (K), sodium (Na), phosphorus (P), magnesium (My), calcium (Ca), lactic acid (Lac), immunoglobulins (IgG, IgM, IgA), activated partial thromboplastin time (APTT), prothrombin time (PT), fibrinogen (Fib), and plasma D-dimer assay (D-dimer), all serological markers were tested within 24 h of admission. The subvariable categorization of PCT was based on the expert consensus stratification for the clinical use of calcitoninogen in emergency medicine (8) (Table 1), PLT, Lac and Fib grouping boundaries were delineated by the 2024 International Consensus Phoenix Scoring Criteria for Sepsis and Septic Shock in Children (9) (Table 1). All data were collected and cross-checked by two trained researchers, with any inconsistencies addressed and rectified. Following data collection, a database was established for subsequent statistical analyses.

**Table 1:** Patient characteristics of the development dataset [*N* (%), mean ± SD].

Characteristics	Training cohort (*n* = 583)			Test cohort (*n* = 251)		
General characteristics	Sepsis (*n* = 149)	No-sepsis (*n* = 434)	*P*-value	Sepsis (*n* = 63)	No-sepsis (*n* = 188)	*P*-value
Gender:						
Male (%)	101 (67.79)	257 (59.22)	0.079	35 (55.56)	118 (62.77)	0.386
Female (%)	48 (32.21)	177 (40.78)		28 (44.44)	70 (37.23)	
Nation:						
Ethnic Han (%)	105 (70.47)	365 (84.10%)	<0.001	55 (87.30)	154 (81.91)	0.426
Minority (%)	44 (29.53)	69 (15.90%)		8 (12.70)	34 (18.09)	
Age, months						
<12 (%)	87 (58.39)	259 (59.68)	0.948	31 (49.21)	112 (59.57)	0.163
12–36 (%)	27 (18.12)	74 (17.05)		13 (20.63)	22 (11.70)	
>36 (%)	35 (23.49)	101 (23.27)		19 (30.16)	54 (28.72)	
Weight, kg	11.07 ± 8.35	11.76 ± 10.15	0.412	13.44 ± 10.31	11.39 ± 8.05	0.155
Dystrophy:						
Yes (%)	32 (21.48)	41 (9.45)	<0.001	10 (15.87)	18 (9.57)	0.253
No (%)	117 (78.52)	393 (90.55)		53 (84.13)	170 (90.43)	
Site of infection						
Lung (%)	52 (34.90)	249 (57.37)	<0.001	23 (36.51)	105 (55.8)	<0.001
Intestinal (%)	33 (22.15)	88 (20.28)		5 (7.94)	42 (22.34)	
Intracranial (%)	43 (28.86)	63 (14.62)		25 (39.68)	24 (12.77)	
Urethra (%)	8 (5.37)	13 (3.00)		2 (3.17)	8 (4.26)	
Other (%)	13 (8.72)	21 (4.84)		8 (12.70)	9 (4.79)	
Clinical characteristics						
Respiratory Rate (beats/mins)						
Low (%)	8 (5.37)	96 (22.12)	<0.001	2 (3.17)	43 (22.87)	<0.001
Normal (%)	89 (59.73)	293 (67.51)		40 (63.49)	128 (68.09)	
High (%)	52 (34.90)	45 (10.37)		21 (33.33)	17 (9.04)	
Heart Rate (beats/mins)						
Low (%)	2 (1.34%)	10 (2.30%)	0.006	0 (0.00)	5 (2.66)	0.015
Normal (%)	85 (57.05%)	304 (70.05%)		32 (50.79)	126 (67.02)	
High (%)	62 (41.61%)	120 (27.65%)		31 (49.21)	57 (30.32)	
Temperature, °C						
<36.0 (%)	4 (2.68)	1 (0.23)	<0.001	3 (4.76)	0 (0.00)	<0.001
36.0–37.3 (%)	45 (30.20)	234 (53.92)		19 (30.16)	109 (57.98)	
37.4–38.5 (%)	58 (38.93)	125 (28.80)		20 (31.75)	54 (28.72)	
>38.5 (%)	42 (28.19)	74 (17.05)		21 (33.33)	25 (13.30)	
ABP, mmHg	91.33 ± 18.66	92.41 ± 13.38	0.514	90.59 ± 19.44	92.09 ± 12.65	0.567
DBP, mmHg	55.97 ± 14.18	55.97 ± 11.95	0.996	54.71 ± 15.8	55.14 ± 11.61	0.845
PP, mmHg	35.36 ± 12.75	36.44 ± 9.32	0.344	35.87 ± 11.35	36.95 ± 9.64	0.500
MAP, mmHg	67.75 ± 14.63	68.12 ± 11.65	0.783	66.67 ± 16.24	67.46 ± 11.07	0.723
Laboratory findings						
WBC*10^9^/L	12.78 ± 9.35	12.21 ± 7.6	0.503	11.98 ± 7.86	12.32 ± 8.43	0.769
L%	32.63 ± 18.42	34.97 ± 20.19	0.193	32.5 ± 23.08	34.31 ± 21.33	0.582
N%	63.8 ± 19.31	54.86 ± 23.24	<0.001	62.79 ± 23.87	55.8 ± 23.54	0.046
IG%	2.31 ± 2.53	0.65 ± 0.95	<0.001	2.74 ± 3.83	0.7 ± 1.45	<0.001
Hb, g/L	107.62 ± 24.06	113.34 ± 29.32	0.019	104.66 ± 23.69	112.84 ± 17.74	0.014
ALB, g/L	35.82 ± 8.08	39.48 ± 6.52	<0.001	34.5 ± 6.88	39.19 ± 5.9	<0.001
TBIL, umol/L	22.59 ± 37.92	14.47 ± 22.02	0.014	17.91 ± 21.82	12.89 ± 13.76	0.090
Glu, mmol/L	5.43 ± 2.7	5.26 ± 1.85	0.467	5.54 ± 3.41	5.23 ± 1.92	0.487
Na, mmol/L	136.25 ± 4.18	135.81 ± 6.35	0.239	135.16 ± 6.16	135.35 ± 4.39	0.817
K, mmol/L	4.36 ± 1.01	4.55 ± 0.82	0.036	4.11 ± 0.91	4.55 ± 0.9	0.001
P, mmol/L	1.65 ± 0.9	1.48 ± 0.42	0.032	1.43 ± 0.73	1.46 ± 0.4	0.802
Ca, mmol/L	2.17 ± 0.35	2.37 ± 0.25	<0.001	2.08 ± 0.37	2.33 ± 0.28	<0.001
Mg, mmol/L	0.94 ± 0.27	0.91 ± 0.15	0.184	0.93 ± 0.19	0.9 ± 0.14	0.274
Cr, umol/L	50.87 ± 68.38	25.62 ± 24.63	<0.001	48.7 ± 47.84	25.18 ± 18.6	<0.001
Cysc, mg/L	1.45 ± 0.92	1.05 ± 0.51	<0.001	1.24 ± 0.67	1.09 ± 0.62	0.142
CRP, mg/L	106.77 ± 115.35	66.02 ± 86.49	<0.001	120.54 ± 113.66	70.47 ± 83.27	0.002
IgG, g/L	5.8 ± 3.75	6.38 ± 3.93	0.109	6.41 ± 3.76	6.3 ± 4.13	0.838
IgM, g/L	0.82 ± 0.66	0.83 ± 0.55	0.881	0.8 ± 0.47	0.9 ± 0.67	0.192
IgA, g/L	0.8 ± 1.24	0.56 ± 0.61	0.020	0.76 ± 0.7	0.58 ± 0.72	0.078
APTT, s	49.21 ± 28.75	37.89 ± 16.96	<0.001	49.6 ± 26.15	37.2 ± 14.87	<0.001
PT, s	18.75 ± 13.27	13.06 ± 5.05	<0.001	20.22 ± 13.97	12.74 ± 2.87	<0.001
D-dimer, mg/L	9.52 ± 15.98	2.29 ± 3.62	<0.001	10.94 ± 14.4	1.98 ± 2.2	<0.001
Fib, g/L						
>1.0 (%)	128 (85.91)	423 (97.47)	<0.001	53 (84.13)	187 (99.47)	<0.001
≤1.0 (%)	21 (14.09)	11 (2.53)		10 (15.87)	1 (0.53)	
Lac, mmol/L						
<5 (%)	111 (74.50)	420 (96.77)	<0.001	51 (80.95)	183 (97.34)	<0.001
5–10.9 (%)	31 (20.81)	14 (3.23)		11 (17.46)	5 (2.66)	
≥11 (%)	7 (4.70)	0 (0.00)		1 (1.59)	0 (0.00)	
PLT*10^9^/L						
≥100 (%)	97 (65.10)	432 (99.54)	<0.001	43 (68.25)	188 (100.00)	<0.001
<100 (%)	52 (34.90)	2 (0.46)		20 (31.75)	0 (0.00)	
PCT, ng/ml						
<0.5 (%)	5 (3.36)	189 (43.55)	<0.001	7 (11.11)	78 (41.49)	<0.001
0.5–2 (%)	18 (12.08)	129 (29.72)		2 (3.17)	47 (25.00)	
2–10 (%)	32 (21.48)	80 (18.43)		15 (23.81)	41 (21.81)	
>10 (%)	94 (63.09)	36 (8.29)		39 (61.90)	22 (11.70)	

### 2.3 Statistical methods

All statistical analyses were performed using R software (version 4.4.0) with dedicated packages, including “glmnet” for LASSO regression, “pROC” for ROC curve analysis, and “rms” for calibration plots. Statistical significance was defined as *P*-values below 0.05.

The distribution of continuous variables was assessed using the Shapiro–Wilk test. Normally distributed continuous variables were expressed as mean ± standard deviation (Mean ± SD)), while categorical variables were expressed as percentages (%) and assessed using the Pearson chi-square or Fisher's exact test.

To mitigate potential issues of multicollinearity and overfitting, variables selection was conducted using the Least Absolute Shrinkage with Selection Operator Algorithm (LASSO) regression analysis method. Through LASSO regression, data dimensionality was reduced by shrinking the coefficients of uncorrelated variables to zero, while retaining variables with non-zero coefficients as potential predictor variables.Initially, all patients were randomly allocated to a training set and a validation set in a 7:3 ratio. Univariate analysis was performed on 39 variables. Variables achieving a significance level of *P* < 0.05 were subsequently incorporated into LASSO regression analysis. A logistic regression model was then developed using the training set. Following model development, the Receiver Operating Characteristic (ROC) curve, calibration curve, and Decision Curve Analysis (DCA) were calculated in both the training set and the validation set. The optimal shrinkage parameter lambda was determined via 10-fold cross-validation using the “cv.glmnet” function within the “glmnet” package. In this study, lambda. 1se (within one standard error of the minimum lambda) was selected as the optimal lambda choice as it facilitates the selection of the minimum number of variables while maintaining good predictive performance.

Predictors identified through LASSO regression were incorporated into a multivariate logistic regression analysis to ascertain independent risk factors associated with sepsis, along with their corresponding regression coefficients (b) and intercept values. Column plots were constructed based on the outcomes of the multivariate logistic regression analysis utilizing the “rms” package in R. Subsequently, patients within the development dataset were randomly split into 70% for training and 30% for internal validation using the “caret” package. The predictive model's clinical efficacy was assessed by evaluating the performance of the column plots in both the training and validation sets through “pROC”, “rms” and “rmda” packages, respectively.

## 3 Results

### 3.1 Comparison of general patient characteristics

A total of 834 children hospitalised for infection were included in the dataset. Table 1 illustrates that males accounted for 61.27% (511/834) of the cohort, while females comprised 38.73% (323/834). There were no statistically significant differences observed in the age and weight distributions between the sepsis and non-sepsis group (*P* > 0.05). However, the proportion of patients in the sepsis group with the nervous system as the primary site of infection was significantly higher compared to the non-sepsis group (28.86% vs. 14.62%, *P* < 0.001).

### 3.2 Comparison of patients' vital signs and laboratory tests

Significant differences were observed in various vital signs and laboratory test results between the sepsis and non-sepsis groups. The sepsis group exhibited a notably higher proportion of respiratory rate and heart rate above the normal range for the corresponding age group compared to the non-sepsis group (34.90% vs. 10.37%, *P* < 0.001), (41.61% vs. 27.65%, *P* = 0.006). Additionally, the sepsis group showed a higher incidence of hypothermia (<36°C) and hyperthermia (>38.5°C) than the non-sepsis group, with statistically significant differences (*P* < 0.001). Regarding laboratory tests, parameters including N%, IG%, Hb, ALB, TBIL, K, P, Ca, Cr, Cysc, CRP, IgA, APTT, PT, D-dimer, Fib, Lac, PLT, and PCT in the sepsis group were significantly different from those in the non-sepsis group (*P* < 0.05) (Table 1).

### 3.3 Variable selection and modelling of column-line plots

**Figure 2:**
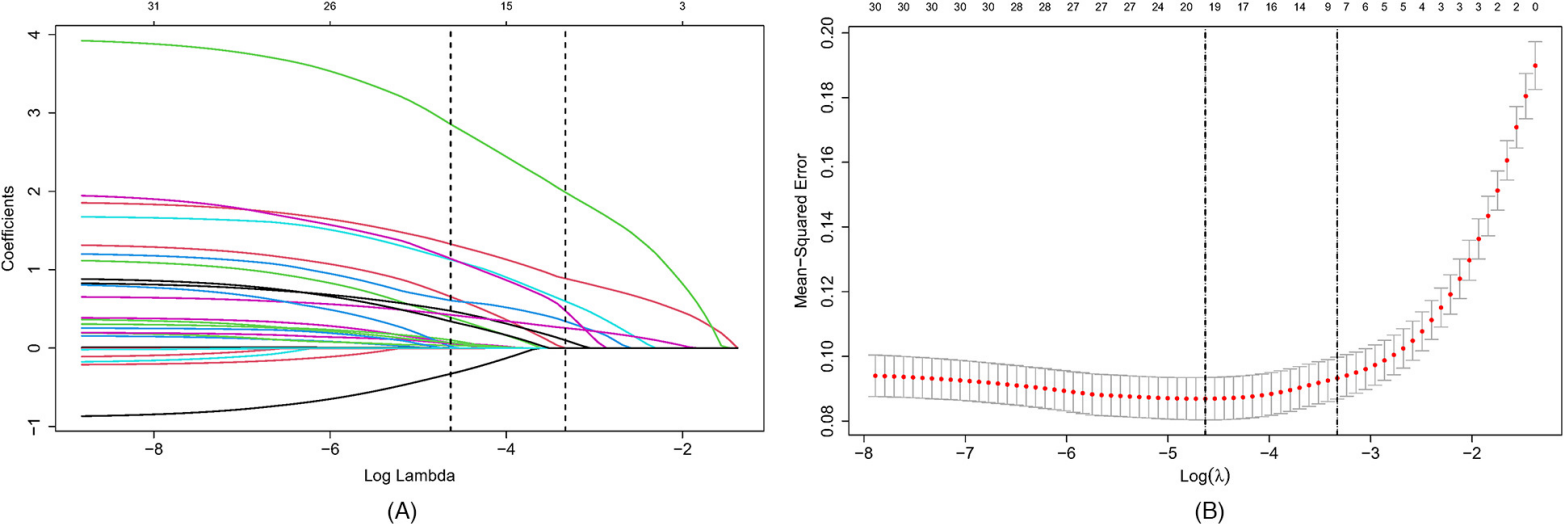
Selection of candidate predictor variables using LASSO regression. **(A)** Distribution of LASSO coefficients for 39 potential predictor variables. **(B)** Optimization of parameter (lambda) selection in the LASSO model using 10-fold cross-validation based on one standard error of the minimum criterion (lambda. 1se). Seven non-zero coefficient variables were selected as candidate predictors. LASSO, minimum absolute shrinkage and selection operator.

**Figure 3:**
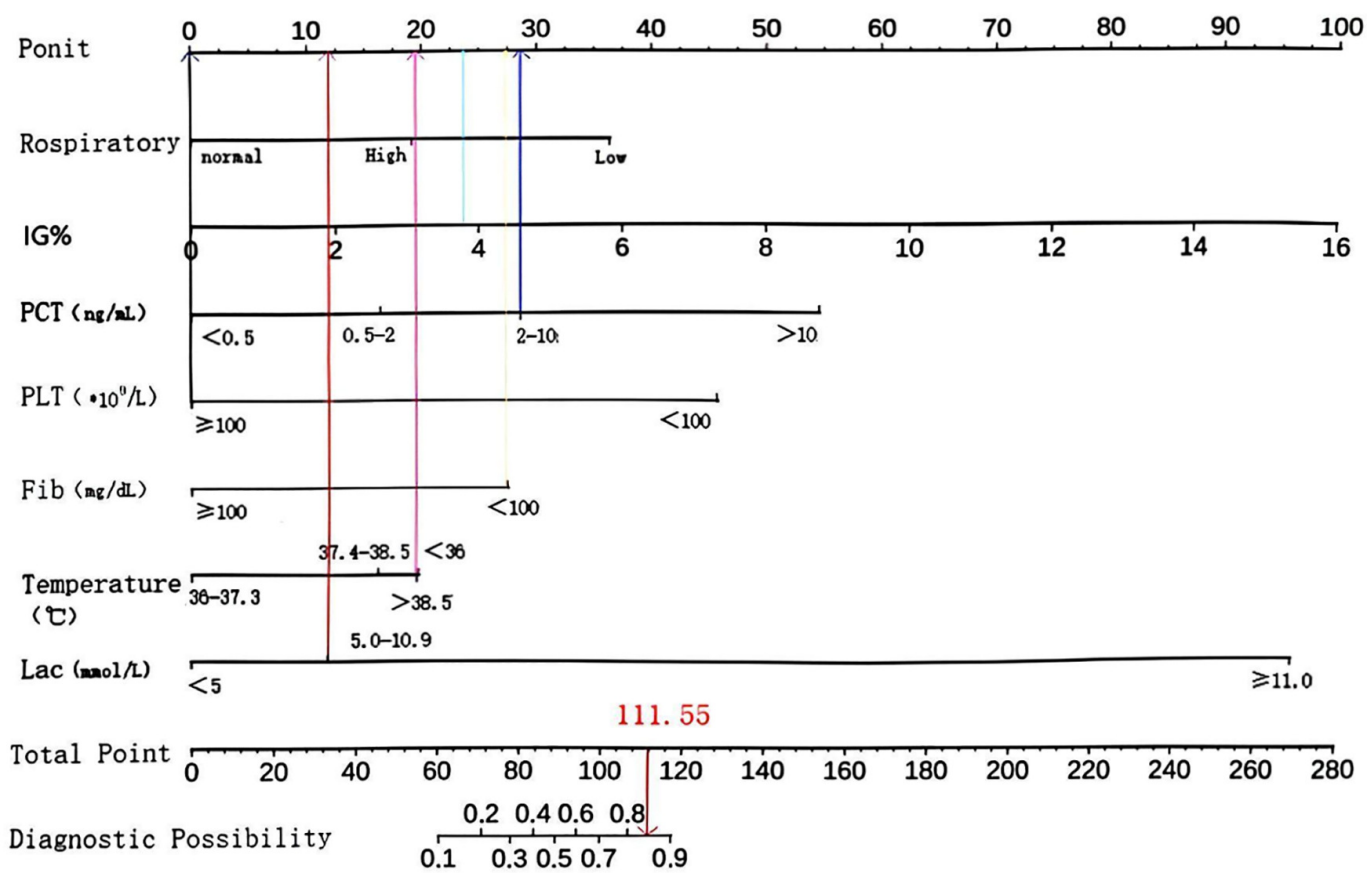
Column line diagram.

The primary objective of this study was to establish a predictive model for pediatric sepsis diagnosis.This model targets sepsis prediction within the first 48 h of hospital admission. A total of 39 variables pertinent to the diagnostic process underwent univariate analysis, revealing that ethnicity, nutritional status, site of primary infection, respiratory rate, heart rate, body temperature, N%, IG%, Hb, ALB, TBIL, K, P, Ca, Cr, Cysc, CRP, IgA, APTT, PT, D-dimer, Fib, Lac, PLT, PCT, 25 variables, were independent risk factors for the development of sepsis (*P* < 0.05) (Table 2). These 25 variables were included in the LASSO regression, with lambda. 1se being chosen as the optimal lambda. Subsequently, seven independent risk factors were identified, including respiratory rate (RR), temperature (T), immature granulocyte percentage (IG%), fibrinogen (Fib), lactic acid (Lac), platelets (PLT), and procalcitonin (PCT) (Figures 2A,B). These seven independent risk factors were then subjected to multifactorial analysis (Table 3). To enhance the intuitiveness and practicality of the predictive model, it was visualized as a column-line graph incorporating the seven independent predictors. Each variable's result on the graph corresponds to its respective test outcome, with the associated predictor score indicated at the top. By summing the scores for each indicator, the total predicted score can be determined, offering insight into the likelihood of sepsis occurrence in the child. For example, for an 8-month-old infant with a respiratory rate of 55 breaths/min (0 points), a temperature of 39°C (19.7 points), immature granulocyte percentage of 3.8% (23.75 points), a procalcitonin (PCT) level of 6 ng/ml (28.6 points), a platelet count (PLT) of 105*10^9^/L (0 points), a fibrinogen (Fib) level of 90 mg/dl (27.5 points), and a lactate level of 6 mmol/L (12 points), the calculated total score would be 111.5, corresponding to a predicted sepsis risk of 0.84 (Figure 3).

**Table 2:** Univariate analysis of early diagnosis of sepsis in children.

Characteristics	B	SE	OR (95% CI)	Z	*P*
Gender:	−0.371	0.20069	0.69 (0.463–1.018)	−1.849	0.065
Nation	0.796	0.22245	2.217 (1.428–3.421)	3.578	0.001
Age	0.022	0.11336	1.022 (0.816–1.273)	0.19	0.849
Weight	−0.008	0.01026	0.992 (0.972–1.012)	−0.747	0.455
Dystrophy	0.964	0.25832	2.622 (1.573–4.343)	3.731	0.001
Site of infection	0.364	0.07776	1.439 (1.236–1.678)	4.68	0.001
Respiratory Rate	1.323	0.1873	3.756 (2.623–5.472)	7.065	0.001
Heart Rate	0.593	0.18914	1.81 (1.25–2.625)	3.137	0.002
Temperature	0.471	0.11934	1.602 (1.268–2.026)	3.946	0.001
ABP	−0.005	0.00643	0.995 (0.983–1.008)	−0.766	0.443
DBP	0	0.00757	1 (0.985–1.015)	−0.005	0.996
PP	−0.01	0.00939	0.99 (0.971–1.008)	−1.101	0.271
MAP	−0.002	0.00764	0.998 (0.983–1.013)	−0.309	0.757
WBC	0.008	0.01144	1.009 (0.986–1.031)	0.741	0.458
L%	−0.006	0.00487	0.994 (0.984–1.003)	−1.247	0.213
N%	0.019	0.00452	1.019 (1.01–1.028)	4.106	0.001
IG%	0.728	0.08737	2.071 (1.758–2.477)	8.332	0.001
Hb	−0.011	0.00453	0.989 (0.981–0.998)	−2.344	0.019
ALB	−0.075	0.01454	0.927 (0.901–0.954)	−5.185	0.001
TBIL	0.009	0.00325	1.009 (1.003–1.016)	2.908	0.004
Glu	0.038	0.0437	1.039 (0.951–1.13)	0.87	0.384
Na	−0.02	0.01684	0.98 (0.948–1.013)	−1.191	0.234
K	−0.266	0.11492	0.766 (0.609–0.956)	−2.316	0.021
P	0.444	0.15481	1.559 (1.153–2.126)	2.87	0.004
Ca	−2.545	0.38438	0.078 (0.036–0.163)	−6.62	0.001
Mg	0.816	0.47893	2.262 (0.874–5.847)	1.704	0.088
Cr	0.019	0.00399	1.019 (1.012–1.028)	4.715	0.001
Cysc	0.927	0.17085	2.528 (1.836–3.586)	5.428	0.001
CRP	0.004	0.00093	1.004 (1.002–1.006)	4.313	0.001
IgG	−0.042	0.02697	0.959 (0.907–1.008)	−1.569	0.117
IgM	−0.027	0.16567	0.973 (0.696–1.336)	−0.165	0.869
IgA	0.33	0.11495	1.391 (1.121–1.762)	2.871	0.004
APTT	0.022	0.00432	1.022 (1.014–1.031)	5.118	0.001
PT	0.092	0.0181	1.096 (1.062–1.14)	5.086	0.001
D-dimer	0.128	0.01972	1.136 (1.096–1.184)	6.481	0.001
Fib	1.842	0.38562	6.309 (3.019–13.9)	4.777	0.001
Lac	2.195	0.32321	8.983 (4.912–17.55)	6.792	0.001
PLT	4.752	0.72898	115.7 (35.18–715.2)	6.518	0.001
PCT	1.502	0.12955	4.492 (3.521–5.858)	11.596	0.001

**Table 3:** Multifactorial analysis of early diagnosis of sepsis in children.

Characteristics	B	SE	OR (95% CI)	Z	*P*
Respiratory Rate	1.383	0.2878	3.985 (2.307–7.160)	4.804	0
Temperature	0.721	0.19924	2.056 (1.400–3.067)	3.619	0
IG %	0.962	0.47434	2.618 (1.056–6.787)	2.029	0.042
Fib	0.465	0.10483	1.592 (1.306–1.972)	4.439	0
Lac	1.446	0.1771	4.247 (3.055–6.136)	8.166	0
PLT	3.586	0.78226	36.09 (9.571–238.6)	4.584	0
PCT,	2.246	0.6997	9.448 (2.403–37.78)	3.21	0.001
Intercept	−12.079	1.35656	5.676 (3.318–6.882)	−8.904	0

### 3.4 Internal validation of column line diagrams

**Figure 4:**
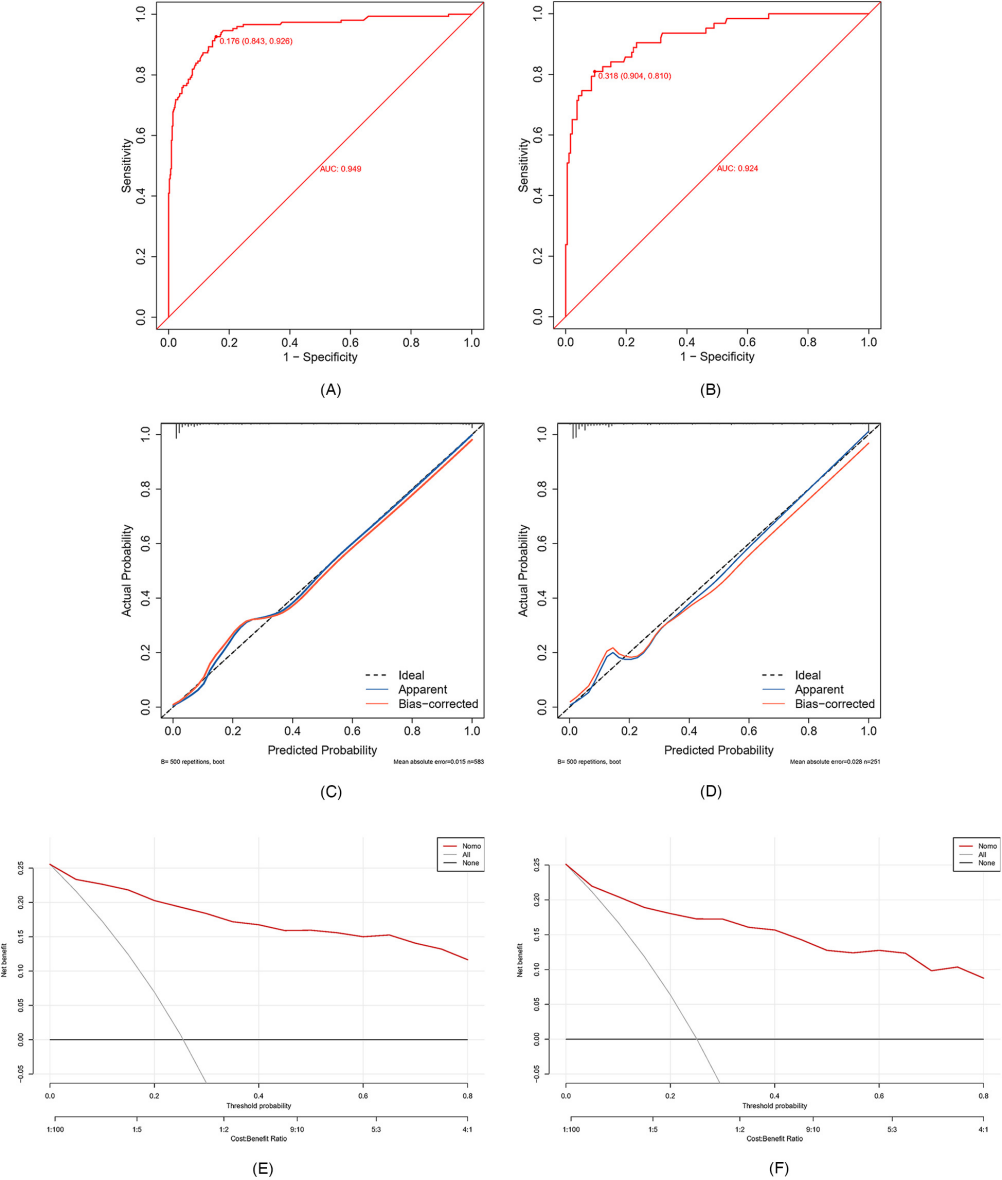
**(A,B)** ROC curves. **(C,D)** Calibration curve. **(E,F)** DCA. ROC curve: subjects’ work characteristic curve; DCA: decision curve analysis.

In this study, patients were divided into a training set and a validation set in a ratio of 7:3. The validation set was used for internal validation to test the predictive performance of the model. The ROC curve for the training set exhibited an AUC of 0.949 (95% CI: 0.927–0.971, *P* < 0.001) with a sensitivity of 92.6%. The specificity was 84.3%, demonstrates good discriminative power for sepsis occurring within 48 h (Figure 4A). Similarly, the ROC curve for the validation set demonstrated an AUC of 0.924 (95% CI: 0.810–0.904, *P* < 0.001) with a sensitivity of 81.0% and specificity of 90.4% (Figure 4B). The column-line plot displayed good calibration based on the Hosmer-Lemeshow test results: training set 2 = 7.9663, *P* = 0.437; validation set 2 = 10.072, *P* = 0.260. The calibration curves were visually depicted (Figures 4C,D), indicating a favorable agreement between the predicted probability of sepsis occurrence in children using the column-line plot and the actual probability of sepsis occurrence, with good calibration in both the training and internal validation sets. A DCA analysis was performed using the threshold probability as the horizontal coordinate and the net benefit rate as the vertical coordinate to evaluate the clinical application value of the column-line diagram. As illustrated in Figures 4E,F, the column chart can provide a greater net benefit than the “all” and “none” scenarios, with the training set (threshold probability: >0.2) and the internal validation set (threshold probability: >0.2), indicating superior clinical utility.

## 4 Discussion

In this study, we developed and validated a new, simple and effective predictive model for the risk of sepsis onset up to 48 h in advance, updated continuously as new data is received. Utilizing fundamental vital signs and key laboratory parameters including respiratory rate, body temperature, percentage of naïve granulocytes, platelet count, procalcitonin, fibrinogen and lactate, column-line diagrams were constructed. The predictive model underwent internal validation, demonstrating strong predictive capabilities, and can serve as a valuable tool for pediatricians in evaluating children at risk of sepsis development.

Given the diverse clinical symptomatology in the early phases of sepsis, the limited specificity of laboratory markers, and the absence of definitive biomarkers, numerous researchers have advocated for combining multiple biomarkers to enhance early sepsis diagnosis through scoring systems. Sakyi (10) et al. conducted a case-control study involving 60 children aged 0–6 years with sepsis, and 30 non-septic children as controls. They identified CRP, PCT, and preprotease (sCD14-ST) as markers with substantial predictive value and as independent predictors of sepsis in children. While the predictive capacity of PCT aligns with our study's findings, we did not identify CRP as an independent predictor of sepsis, potentially attributed to the limited case numbers in their investigation. Several studies have indicated an association between preprotease (sCD14-ST) and sepsis development (11–13). However, the limited availability of presepsin testing, particularly in primary care settings, significantly hampers the practical application of the model. Our study's strengths lie in the ample sample size, well-defined variables, and the accessibility of their corresponding data. For instance, platelet count and immature granulocyte percentage can be obtained from CBC analyses, while lactate levels can be assessed through arterial blood gas (ABG) analyses. These routine laboratory tests are widely accessible in healthcare facilities across various settings. Florian's (14) team also developed a pediatric sepsis prediction model incorporating laboratory measures of IL-6, platelet count, PCT, CRP, and four clinical parameters including PICU length of stay, presence of central venous catheter, body temperature, and cumulative number of sepsis and SIRS episodes prior to diagnosis. The complexity of the variables in this model may hinder early diagnosis, for instance, PICU length of stay could impede sepsis identification in non-ICU settings. Moreover, the cumulative number of sepsis and SIRS episodes may not be easily interpreted by individuals with low literacy levels. In terms of early and accurate diagnosis, our predictive model surpasses the former. Other researchers have utilized sepsis-related genes to design diagnostic prediction models (15–18), but these models are primarily applicable in large, well-equipped hospitals. In less-developed regions and primary care settings, delayed sepsis diagnosis and elevated mortality rates are more prevalent.

Three of the seven variables screened in this study, including calcitoninogen, platelet count and lactate, are widely acknowledged for their strong association with sepsis diagnosis and severity, as supported by existing research. Platelets are crucial in diagnostic criteria such as the SOFA score and the newly proposed Phoenix score (9). Lactate holds significance in the Phoenix score. The lactate categorization in this study adhered to the latest Phoenix score criteria to ensure broad recognition. PCT exhibits rapid responsiveness post-bacterial infection, typically rising 2–4 h after sepsis onset (19). With its high sensitivity and specificity, PCT serves as a valuable biomarker for sepsis diagnosis and prognostication of disease severity (20). In the non-infected state of the organism, PCT concentrations <0.05 ng/ml are observed, while concentrations exceeding 10 ng/ml suggest severe sepsis or septic shock (8, 21, 22). In a randomized controlled trial by Ali et al. (23), PCT was found to exhibit higher accuracy, specificity, and sensitivity compared to CRP in sepsis diagnosis (80.79% vs. 69.45%, 36% vs. 28.7%, 87.6% vs. 72.4%). In a study by Doerﬂinger et al. (24) focusing on an early prediction model for pediatric sepsis diagnosis using procalcitonin and interleukin-10, the combination of PCT (≥0.425 ng/ml) and IL-10 (≥4.37 pg/ml) demonstrated a sensitivity of 100% (95% CI: 68.8%–100%) and a specificity of 89% (95% CI 80.0–95.0%). This study reported an 11-fold increase in PCT levels in the sepsis group compared to the non-sepsis group, aligning with the findings of the current investigation.

The present study also identified respiratory rate and body temperature as predictors of sepsis in children. The concept of SIRS was introduced as early as 1991 in sepsis 1.0 (25), indicating that a clinical response to non-specific injury (temperature > 38°C or <36°C; heart rate > 90 bpm; respiratory rate > 20 bpm; total white blood cell count > 12*10^−9^/L or <4*10^−9^/L, or proportion of immature (rod-shaped nuclei) neutrophils > 10%) meeting two criteria plus a suspected infection is adequate for sepsis diagnosis. Similarly, the diagnostic criteria of sepsis 2.0 in 2001 included assessment of respiratory rate and temperature (26), although these criteria were later deemed to lack specificity and are no longer in use. Nevertheless, they can still reflect the body's inflammatory response status and criticality level to some extent. Temperature changes can be influenced by various factors, including feeding, environmental temperature, and the level of immune stimulation, as demonstrated in animal studies (27, 28). Severe infections tend to induce more significant and varied temperature fluctuations compared to other causes. A large prospective study showed (29) a bimodal distribution of body temperatures in septic patients, with the development of sepsis linked to hyperthermia and increased mortality associated with hypothermia. This implies that hyperthermia and hypothermia may indicate a more severe and an excessive immune response. Consistent with the findings of this study, a body temperature <36°C or >38.5°C also resulted in a single high score of 20.9. Respiratory dysfunction can cause hypoxemia and dyspnea, with respiratory rate serving as an indicator of respiratory distress (30). Following the onset of sepsis, the respiratory system is commonly affected, and studies have indicated a significant association between abnormal respiratory rate and sepsis diagnosis (31).

Monitoring both temperature and respiratory rate is routine and easily accessible. In this study, alongside assessing sepsis likelihood using respiratory rate and body temperature status, we also combined PCT, PLT, and lactate with other laboratory indicators that are closely related to the development of sepsis to make a comprehensive determination, which is a highly effective guide for the identification of sepsis in children.

Sepsis and septic shock can lead to sepsis-induced coagulation dysfunction by activating inflammatory mediators and causing damage to endothelial cells (32). These fibrinolytic changes induced may lead to microthrombosis and microcirculatory disturbances, ultimately leading to organ failure and increased mortality (33). Fibrinogen plays a key role in haemostasis and antimicrobial host defence (34). In patients with sepsis, fibrinogen levels are often decreased and are associated with a poor prognosis (32). Signoff et al. (35) observed that children with sepsis combined with hypofibrinogenemia may have a more complicated disease course (73.7% vs. 29.2%; *P* < 0.001) and a higher 28-day mortality rate (26.3% vs. 7. 1%, *P* = 0.002). A retrospective study demonstrated a significant association between fibrinogen levels below 2 g/L upon PICU admission and an elevated risk of mortality in pediatric sepsis patients (36). The immature granulocyte percentage, often underappreciated in clinical settings, may rise in infected individuals, particularly in the presence of bacterial infections, as immature naive granulocytes are released prematurely into the peripheral blood. In a study on naive cells as the earliest biomarkers of sepsis, it was mentioned that absolute and immature granulocyte percentage serve as the earliest discriminators of sepsis (37). These markers exhibit an area under the curve of 0.81 and 0.82, respectively, and can be elevated up to 24 h prior to sepsis diagnosis. Therefore, absolute and immature granulocyte percentage represent readily accessible and cost-effective markers with high specificity and sensitivity for distinguishing septic from non-septic patients. A prospective study by Nierhaus et al. (38) similarly found that IG% could differentiate between infected and non-infected patients (*P* < 0.0001) with a sensitivity of 89.2% and a specificity of 76.4%, especially within 48 h following sepsis onset.

In this study, a set of seven variables closely associated with sepsis development and readily accessible were chosen to construct a column chart and an online calculator. To guarantee clinical reliability and practical applicability, the web-based interactive calculator is currently undergoing final stages of optimization and validation testing. Due to the complexity of consciousness impairment in children with severe sepsis, assessment cannot rely solely on the Glasgow Coma Scale (GCS) and delirium screening tools (39). Moreover, clinical evaluation of consciousness status demonstrates high variability and subjectivity, particularly considering age-related differences (40), Therefore, consciousness assessment was excluded from these variables. The column chart created in this study, based on seven quantifiable indicators, enhances clinical applicability and holds significant potential for reducing child mortality and disability, particularly in resource-limited settings. It should be emphasized that this model is primarily designed for sepsis screening in children upon hospital admission or during clinical deterioration. This approach may have limited clinical utility in children definitively diagnosed with sepsis and concomitant multiple organ dysfunction syndrome (MODS) in the PICU. While the model demonstrates strengths in operational utility and broad applicability, it is essential to acknowledge its limitations, for instance, the single-center retrospective design and lack of external validation may introduce bias in the study. Future endeavors should include external validation or larger prospective studies to enhance the accuracy and generalizability of the prediction model.

## Data Availability

The original contributions presented in the study are included in the article/Supplementary Material, further inquiries can be directed to the corresponding author.
